# Impact of simulated microgravity in short-term evolution of an RNA bacteriophage

**DOI:** 10.3389/fmicb.2025.1680651

**Published:** 2025-12-04

**Authors:** Alicia Rodríguez-Moreno, Sergio Martín-Blázquez, Unai López de Heredia, Álvaro Soto, Ester Lázaro

**Affiliations:** 1 Centro de Astrobiología (CAB), CSIC-INTA, Madrid, Spain; 2 Escuela de Doctorado, Universidad Autónoma de Madrid, Centro Estudios Postgrado, Madrid, Spain; 3 GI en Desarrollo de Especies y Comunidades Leñosas (WooSp), Dpto. Sistemas y Recursos Naturales, ETSI Montes, Forestal y del Medio Natural, Universidad Politécnica de Madrid, Madrid, Spain

**Keywords:** bacteriophage Q**β**, experimental evolution, simulated microgravity, phage-host interactions, space microbiology, molecular evolution

## Abstract

**Introduction:**

Microgravity is a critical environmental factor in space that can alter microbial physiology and virus–host interactions. Understanding these effects is essential for planetary protection and crew health during long-term missions. Bacteriophage Qβ, an RNA virus infecting Escherichia coli F+ strains, provides a relevant model due to its potential presence in the human gut microbiome and its well-characterized evolutionary dynamics.

**Methods:**

We simulated microgravity using a custom-built 3D-clinostat and compared Qβ infections in semisolid medium under standard gravity and simulated microgravity. Twelve evolutionary lines were propagated for ten serial transfers under four experimental conditions combining bacterial growth and infection environments. Viral titers were quantified by plaque assay, and consensus sequences were determined by Sanger sequencing.

**Results:**

Initial infections under simulated microgravity yielded significantly lower viral titers than those in standard gravity, likely due to hindered phage diffusion and delayed infection initiation. After ten transfers, mutation C2011A (amino acid substitution T222N in the A1 virus protein) was fixed in all lines evolved under simulated microgravity but remained absent or polymorphic in standard gravity lines. Under simulated microgravity, the mutation increased virus titers and promoted faster initiation of infections in semisolid medium. However, those effects were not appreciable in normal gravity.

**Discussion:**

Our findings highlight the adaptability of Qβ and the potential impact of microgravity on phage-host interactions, offering insights into virus evolution in extraterrestrial conditions and its implications for space missions and planetary protection.

## 1 Introduction

The harsh conditions of outer space -including extreme temperatures, high levels of radiation and microgravity- pose significant challenges to life as we know it (Horneck, 1981; Horneck et al., 2010; Fukunaga, 2020; Furukawa et al., 2020). Although early studies have explored the ability of diverse organisms to survive in space (Antipov et al., 1967; Thimann, 1968; de Serres, 1969; Taylor, 1974; Klaus et al., 1997; Brown, 1999), much remains unknown about the genetic or phenotypic changes they may undergo when exposed to this environment. In recent years, the growing prospect of long-term and increasingly distant space missions has brought into focus several aspects of this topic that deserve to be investigated in detail.

In the case of unmanned missions, especially those targeting potentially habitable environments such as Mars or the icy moons of the solar system, it is crucial to ensure that the places to be explored remain uncontaminated by terrestrial microorganisms. To achieve this goal, planetary protection protocols are implemented on Earth that limit microbial presence on spacecrafts (McKaig et al., 2024). However, it is equally important to know whether microorganisms can survive the extreme conditions of space travel and the environments being explored. For manned missions, including extended stays on the International Space Station (ISS), additional concerns arise due to the fact that the space environment can significantly impact the physiology and immune system of astronauts (Sonnenfeld, 1998; Aponte et al., 2006; Kuzichkin et al., 2022), potentially increasing their susceptibility to infections caused by viruses and bacteria. Since astronauts aboard the ISS and spacecrafts are shielded from extreme temperatures and radiation but not from microgravity, this condition takes on considerable interest. Gravity has also the peculiarity that has remained constant on Earth since the formation of the planet, meaning that all terrestrial life has evolved without the need to adapt to changes in gravitational force. This fact highlights the importance of investigating how biological systems respond when confronted with gravity conditions to which they have never been exposed.

Changes in gravity can significantly impact biological processes, particularly those involved in the assembly of macromolecular structures (McPherson and DeLucas, 2015; Koaykul et al., 2019; Zhou et al., 2020; Martirosyan et al., 2022). For example, studies conducted with purified VP1 protein, the major capsid protein of polyomavirus, revealed distinct assembly behaviors under different gravity conditions. Ground-based experiments demonstrated that VP1 assembled into capsid-like structures alongside a heterogeneous array of capsomere subunits. In contrast, under microgravity conditions, capsomeres were of uniform size, and no capsid-like structures were formed (Chang et al., 1993). Similarly, the cell-free assembly of bacteriophage T7 virions was enhanced under simulated microgravity compared to static controls (Lehr et al., 2024). Microgravity also influences the growth, survival, and physiology of microorganisms, which can exhibit altered motility, chemotaxis, and cell morphology (Nickerson et al., 2004; Rosenzweig et al., 2010; Rea et al., 2016; Huang et al., 2018; Acres et al., 2021; Cortesão et al., 2022). Of particular importance to human health are the changes in virulence (Wilson et al., 2007; Rosenzweig et al., 2010; Gilbert et al., 2020; Su et al., 2021; Wazir et al., 2023), antibiotic resistance (Mortazavi, 2019; Tirumalai et al., 2019), and host-pathogen interactions (Higginson et al., 2016; Barrila et al., 2021, 2022; Huss et al., 2023). In some cases, the phenotypic changes appear to be linked to altered gene expression, while in others, they may result from disruptions in molecular transport mechanisms (Arunasri et al., 2013; Topolski et al., 2022).

When talking about the risks of microgravity conditions on human health, it is worth noting that our bodies harbor approximately the same number of bacterial as human cells (Sender et al., 2016). These bacteria, which constitute the so-called microbiome, are essential as they are involved in fundamental processes such as immune system regulation, food digestion, and the production of certain neurotransmitters (Gilbert et al., 2018). This means that to evaluate the health risks associated with space missions, it is critical to understand how the components of the microbiome are affected in this environment (Saei and Barzegari, 2012; Cervantes and Hong, 2016; Voorhies and Lorenzi, 2016; Mahnert et al., 2021; Siddiqui et al., 2021). The human microbiome is complex and highly diverse. To function properly, the different species that make it up must maintain an appropriate balance between them, something that is largely regulated by the presence of bacterial viruses (Shkoporov and Hill, 2019). While phage-host interactions have been thoroughly explored in terrestrial ecosystems, their dynamics under microgravity conditions remain unexplored. A recent study carried out with bacteriophage T7 and *Escherichia coli* onboard the ISS identified several mutations in both phage and bacteria that improved fitness in microgravity (Huss et al., 2023). These findings highlight the importance of conducting similar studies with other bacteriophage-host systems to gain a broader understanding of how microgravity influences these interactions.

In this work, we focus on the interaction between the bacterium *E. coli* and one of its specific viruses, the bacteriophage Qβ, which infects strains expressing conjugative pili (F+ strains). Qβ was first isolated in 1961 from human feces (Arisaka, 2024), suggesting its potential presence in the gut environment. Although this does not necessarily imply that it is a stable or abundant component of the intestinal microbiome, its tropism for F+ strains of *E. coli*, which are frequently found in the human gut, makes it a relevant model for studying phage-host interactions in this context. *E. coli* has also served extensively as a model organism in space biology, with prior studies showing that simulated microgravity alters its growth and gene expression (Arunasri et al., 2013; Topolski et al., 2022). These features underscore its suitability for investigating virus–bacteria interactions under conditions that mimic spaceflight.

Using the bacteriophage Qβ-*E. coli* system as an experimental model, we have established a serial transfer protocol that allows us to investigate the evolution of the virus under simulated microgravity conditions in a 3D-clinostat. Qβ belongs to the family *Fiersviridae* (formerly named *Leviviridae*), genus Qubevirus (Walker et al., 2021). The phage has a single-stranded, positive-sense RNA genome of 4,217 nucleotides that encodes four proteins: the A2 protein, which interacts with the cell receptor (the F pilus) and is also involved in the lysis of the infected bacteria (Bernhardt et al., 2001; Cui et al., 2017); the coat protein, the main structural component of the capsid; the A1 protein, a low-abundance capsid protein produced via stop codon readthrough of the coat protein gene (Hofstetter et al., 1974); and the replicase (Kidmose et al., 2010), essential for RNA genome replication. Like other RNA viruses, Qβ replicates with high error rates (Bradwell et al., 2013), resulting in highly heterogeneous populations that can rapidly adapt to a wide variety of environmental conditions. Over the years, we have conducted a number of successful studies concerning the adaptation of this virus to diverse selective pressures (Lázaro et al., 2018; Somovilla et al., 2019; Laguna-Castro et al., 2023, 2024). However, this is the first time when the selective pressure assayed - simulated microgravity - is not a condition found naturally on Earth.

The main objectives of the current work were to investigate how microgravity conditions, simulated in a 3D-clinostat, affect the yield of Qβ infections and to identify the genetic and phenotypic changes experienced by the virus when evolving in this environment. Our results show that the virus titers obtained in infections carried out in the 3D-clinostat were lower than those achieved in a standard incubator. Nevertheless, the virus was able to improve its performance in the simulated microgravity environment within a short period, showing its extraordinary capacity to adapt to new conditions. Studies of this type are crucial for understanding the effects of the space environment on microorganisms, not only to ensure the safety and success of space missions but also to advance our ability to explore new worlds, where the limits of terrestrial biology may be challenged and redefined.

## 2 Materials and methods

### 2.1 Description of the 3D-clinostat

To grow bacteria and perform virus infection under altered gravity, we designed and constructed a 3D-clinostat with two rotation axes, which allow us to avoid a predominant directionality with respect to the culture.

**Figure 1:**
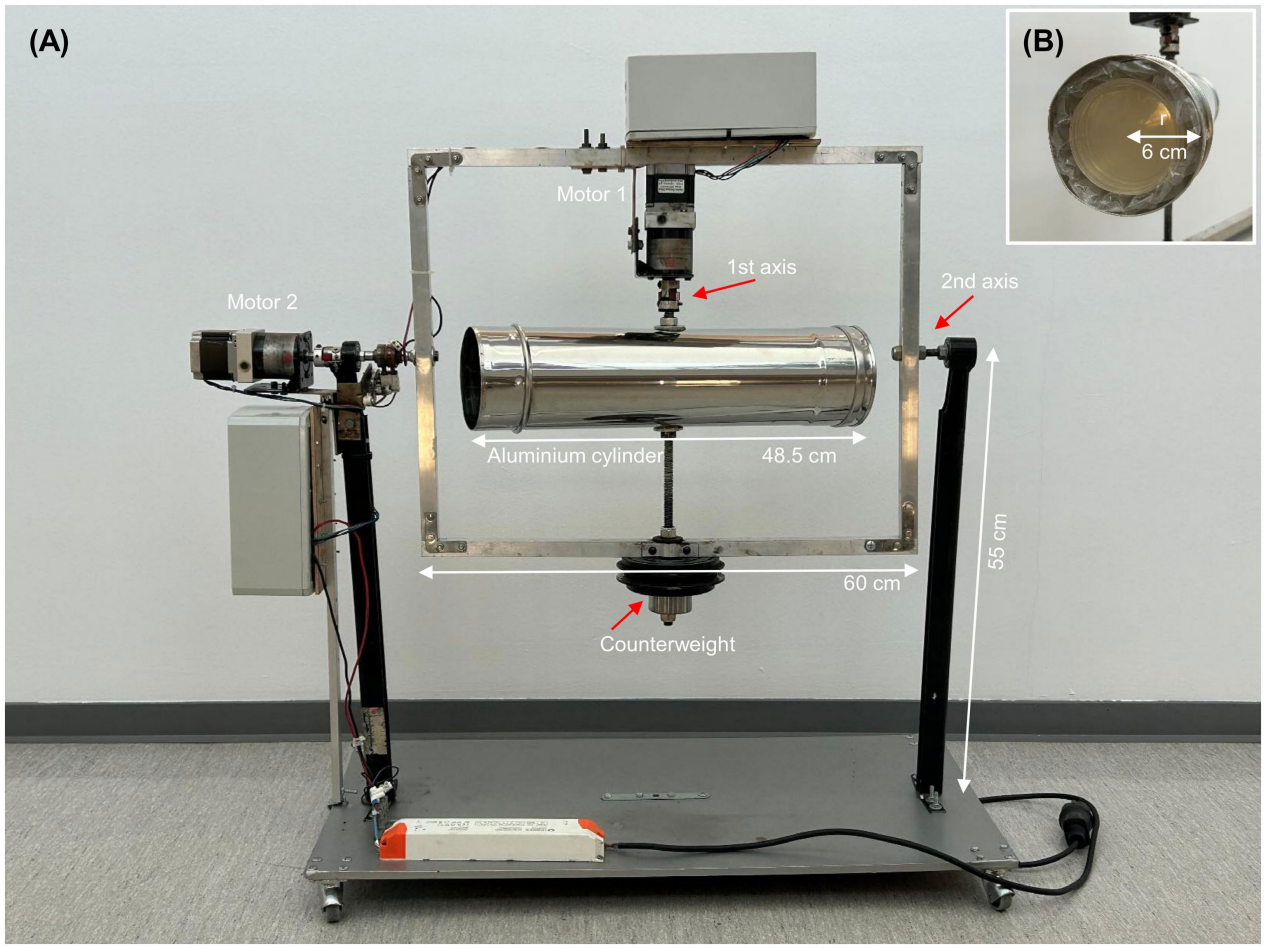
Design and dimensions of the 3D-clinostat. **(A)** Photograph of the custom-built 3D-clinostat, consisting of an aluminum cylinder (48.5 cm long, 6 cm radius) mounted on a rectangular frame that rotates around two perpendicular axes. Two independent motors drive rotation at adjustable speeds, enabling the cylinder to traverse all orientations on a sphere and simulate microgravity conditions. Dimensions of the main components are indicated. **(B)** Detailed view of the cylinder’s dimensions and configuration. Up to 16 Petri dishes can be stacked inside the cylinder, and culture tubes can be inserted longitudinally along its lateral surface.

The clinostat consists of a 48.5 cm-long aluminum cylinder, inside which up to 16 Petri dishes can be stacked (Figure 1). The cylinder is attached to a rectangular frame by means of a steel bar, perpendicular to the cylinder longitudinal axis through its middle point, and which serves as first rotation axis. The frame rotates, in turn, around a coplanar second axis, perpendicular to the first one, which it intersects in the center of the cylinder. For liquid medium cultures, culture tubes were fixed longitudinally on the lateral surface of the cylinder, evenly distributed.

Two separate motors drive rotation around each axis. Rotation speed can be adjusted independently, using an Arduino Uno plate and motor driver shield (Arduino, Italy) for each one, forcing the cylinder to go through all positions on a sphere (Kim et al., 2017; Clary et al., 2022). We finally chose 3:2.73 rpm (first:second axes) to obtain the desired trajectory, maintaining negligible centrifugal effects. Simulated microgravity (estimated as the centrifugal force due to rotation around the first axis, the only effort applied constantly in the same direction with respect to the culture plates) ranged between 3 × 10^−4^ g (innermost position in the cylinder) and 2.4 × 10^−3^ g (outermost position), consistent with previously reported values for bacterial cultures under similar conditions (Arunasri et al., 2013; Topolski et al., 2022). The trajectory was verified with an accelerometer and gyroscope MPU6050 (Arduino, Italy) on an Arduino Uno Rev. 3 plate fixed on the surface of the cylinder (Supplementary Figure 1).

### 2.2 Viruses and bacteria

The plasmid pBRT7Qβ, which contains a cDNA of bacteriophage Qβ cloned in the plasmid pBR322 (Taniguchi et al., 1978; Barrera et al., 1993), was used to transform *E. coli* DH5-*α*, a strain that permits virus expression, although it cannot be infected because it lacks the virus receptor. The supernatant of an overnight culture obtained from a transformed colony was used to infect *E. coli*, strain Hfr (Hayes, 1953), in semisolid agar. The virus progeny contained in a randomly chosen lysis plaque was isolated, and 10^6^ plaque forming units (pfu) were used to infect an *E. coli* Hfr culture under the standard conditions used in our laboratory (37 °C, 250 rpm for 2 h in NB medium: 8 g/L Nutrient Broth from Merck and 5 g/L NaCl) in a New Brunswick Scientific Innova 42 Incubator Shaker (Eppendorf, Enfield, CT, USA), which will hereafter be referred to as the standard incubator. The culture was treated with chloroform (1/20 v/v, 28 °C, 15 min, shaking 850 rpm in thermoblock) and centrifugated at 13,000 rpm. The supernatant containing the virus particles was used as the ancestor of all the evolutionary lines analyzed in this work. It was denoted Qβ_Anc_ and its consensus sequence showed no mutations relative to the Qβ cDNA cloned in pBR322.

The plasmid pBRT7Qβ was also used to engineer a single-mutant virus (Qβ_C2011A_) containing the mutation C2011A. The protocol to obtain this mutant has been previously described (Laguna-Castro and Lázaro, 2022).

Bacteria used for infections (*E. coli* Hfr) were obtained by inoculating a well-isolated colony in a 12 mL polystyrene tube (deltalab) containing 2 mL of NB medium that was incubated for 7 h at 37 °C either in the standard incubator above described (vertical position; 250 rpm) or in the 3D-clinostat (placed in a thermally regulated room). The stationary phase cultures thus obtained were diluted 1:20 in similar tubes until completing a volume of 2 mL in NB medium, and incubated in the same incubator where they had reached the stationary phase as long as necessary to reach an OD_600_ around 0.8. The exponential phase bacteria thus obtained were infected with the phage in the conditions described in each experiment.

### 2.3 Standard procedures for infections in semisolid medium

Infections in semisolid medium were carried out by mixing 300 μL of the corresponding exponential phase bacteria with 100 μL of phage buffer (1 g/L gelatin, 0.05 M Tris–HCl, pH 7.5, and 0.01 M MgCl_2_) containing the indicated pfu and 4.5 mL of melted top agar (7 g/L agar in NB medium). This mix was poured onto Petri dishes containing a bottom agar layer (32 g/L LB Agar, Lennox L Broth Base from Invitrogen). The Petri dishes were incubated for 2 h at 37 °C in horizontal position without shaking (standard incubator) or in the 3D-clinostat (see Figure 1B). After this time, 4 mL of phage buffer was added to each plate and kept shaking horizontally (110 rpm) for 20 min at 25 °C. The buffer was transferred to eppendorf tubes and centrifuged at 13,000 rpm for 10 min to collect the supernatant containing the virus particles, which were maintained at 4 °C for short-term use (less than 15 days) or at −80 °C for long-term storage.

In all cases, virus titers were determined by plaque assay and expressed as the number of pfu per mL of the phage suspension.

### 2.4 Evolution experiment

**Figure 2:**
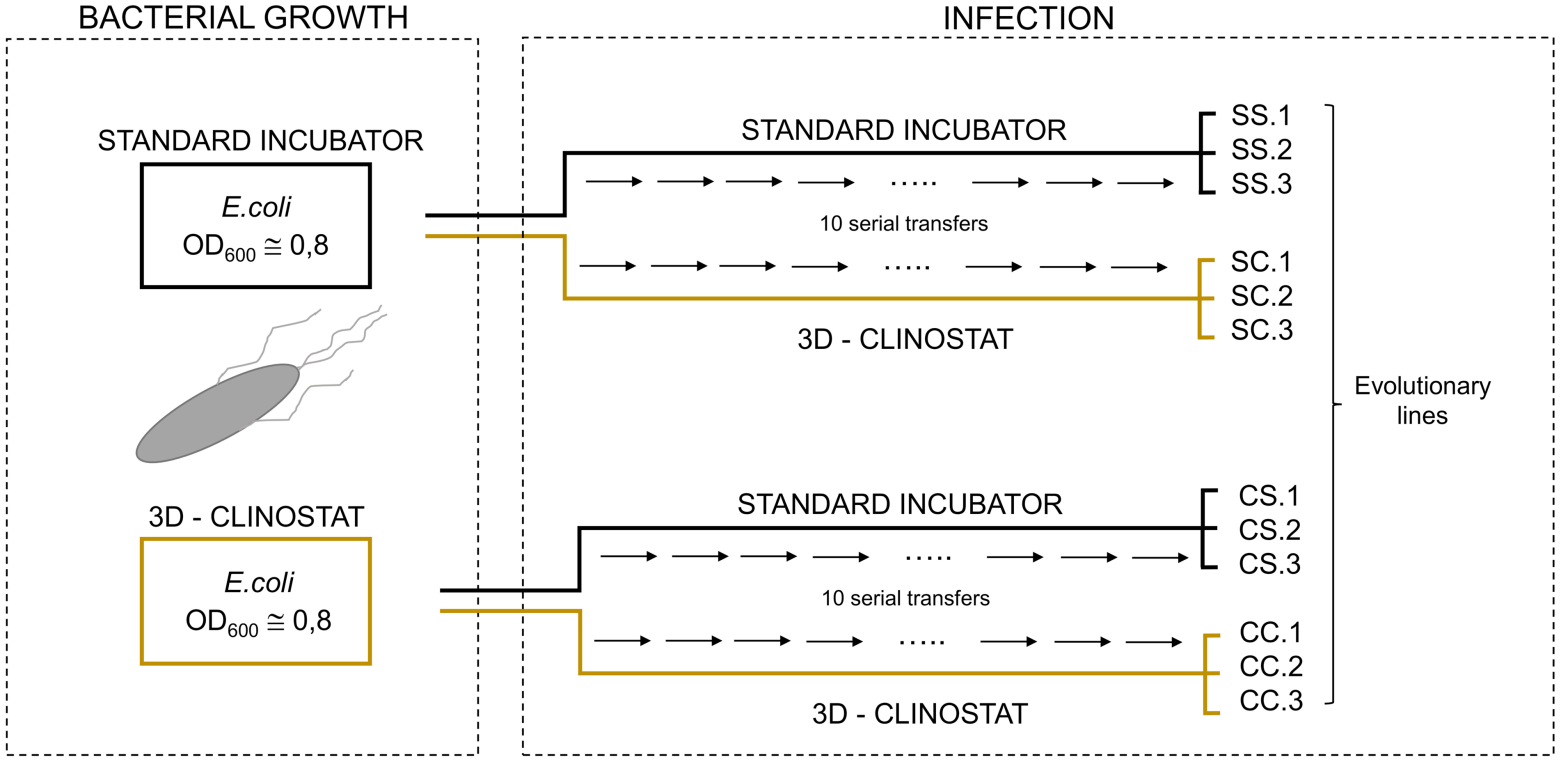
Scheme of the evolution experiment. Two *E. coli* cultures were grown in liquid medium to an OD_600_ ≅ 0.8, either in the standard incubator (S) or in the 3D-clinostat (C). Each culture was then used to initiate six evolutionary lines, where phage propagation occurred in semisolid medium: three lines were incubated under standard conditions and three in the 3D-clinostat. The resulting evolutionary lines were designated as SS, SC, CS, and CC, followed by a number (1, 2, or 3) to identify each replicate. In this nomenclature, the first letter refers to the growth condition of the bacteria prior to infection, and the second letter indicates the condition under which the infection took place. For further details, refer to the “Evolution experiment” section in Materials and methods.

The virus Qβ_Anc_ was used to initiate 12 evolutionary lines that were propagated for 10 serial transfers in semisolid medium (Figure 2). Prior to infection, bacteria were grown in liquid medium, either in the standard incubator or in the 3D-clinostat until an OD_600_ of around 0.8. Infections were carried out in semisolid medium as described in the above section. We defined four experimental conditions that were assayed in triplicate: bacteria grown and infected in the standard incubator (SS lines), bacteria grown in the standard incubator and infected in the 3D-clinostat (SC lines), bacteria grown in the 3D-clinostat and infected in the standard incubator (CS lines), and bacteria grown and infected in the 3D-clinostat (CC lines). In all cases, infection at the first transfer was performed with 10^7^ pfu of the ancestral virus (virus Qβ_Anc_; see Section 2.2). Subsequent transfers until completing a total of 10 were performed with 10^7^ pfu of the virus supernatant obtained at the previous transfer of each line. All transfers were performed after a standardized incubation time of 2 h, regardless of the condition (standard gravity or simulated microgravity). This decision was based on previous experience with Qβ infections in semisolid medium and aimed to maintain consistency across all experimental lines.

Two negative controls in which bacteria were incubated in the absence of virus, both in the standard incubator and in the 3D-clinostat, were set at each transfer. These controls were processed and plated exactly the same as the experimental samples, and were run in parallel using the same equipment and conditions. Their purpose was to detect any contamination, which could occur either during the preparation of the bacterial cultures or during the course of the experiment. When lysis plaques appeared in a control plate, the corresponding transfer was discarded and repeated.

### 2.5 Determination of the virus replicative ability in semisolid medium

The virus yield obtained in replication assays carried out in semisolid medium was used as a measure of the virus replicative ability. For this purpose, infections were performed with 10^4^ pfu of the virus population assayed. We used this amount of virus to ensure that the replicative capacity of the system was not saturated. After 2 h of incubation either in the standard incubator or the 3D-clinostat, virus supernatants were collected and titrated as described above.

### 2.6 Plaque timing experiments

To evaluate the onset of phage-induced lysis under different gravity conditions, we performed plaque timing assays using low viral loads of either the ancestral virus Qβ_Anc_ or the mutant Qβ_C2011A_. Infections were carried out in semisolid agar and incubated either in the standard incubator or in the 3D-clinostat. Lysis plaques were counted at various time points over a 24-h period. For each Petri dish, the percentage of plaques observed at a given time was calculated relative to the total number of plaques counted at 24 h. This normalization allowed for direct comparison of plaque formation kinetics across conditions and viral genotypes. In a separate assay, the number of lysis plaques formed after 4 h of incubation was quantified in five Petri dishes incubated in parallel. The results were also expressed as the percentage of the total plaques observed at 24 h.

### 2.7 RNA extraction, cDNA synthesis, PCR amplification, and nucleotide sequencing

Viral RNA was prepared following standard procedures to determine the consensus sequence either from biological clones or from complex virus populations. RNAs were used for cDNA synthesis with the avian myeloblastosis virus reverse transcriptase (Promega), followed by PCR amplification using Expand high-fidelity DNA polymerase (Roche). The pairs of oligonucleotide primers used for RT-PCR were the following: P1 forward (5′CTTTAGGGGGTCACCTCACAC3′) with P1 reverse (5′GGATGGGTCACAAGAACCGT3′) to amplify from nucleoide position 10 to 1595, P2 forward (5′GACGTGACATCCGGCTCAAA3′) with P2 reverse (5′CAACGGACGGAACATCTCCT3′) to amplify from nucleotide position 1109 to 2787 and P3 forward (5′GTGCCATACCGTTTGACT3^′^) with P3 reverse (5^′^GATCCCCCTCTCACTCGT3^′^) to amplify from nucleotide position 2254 to 4195. PCR products were column purified (Qiagen) and subjected to standard Sanger sequencing using Big Dye Chemistry (v3.1) with an automated sequencer (Abi 3730 XL, Applied Biosystems, Perkin Elmer). Sequences were assembled and aligned with Geneious Pro v4.8.5.^1^ Mutations relative to the sequence of the Qβ cDNA present in the plasmid pBRT7Qβ (virus Qβ_Anc_) were identified using the same software.

## 3 Results

### 3.1 Experimental setup and bacterial growth under simulated microgravity

**Figure 3:**
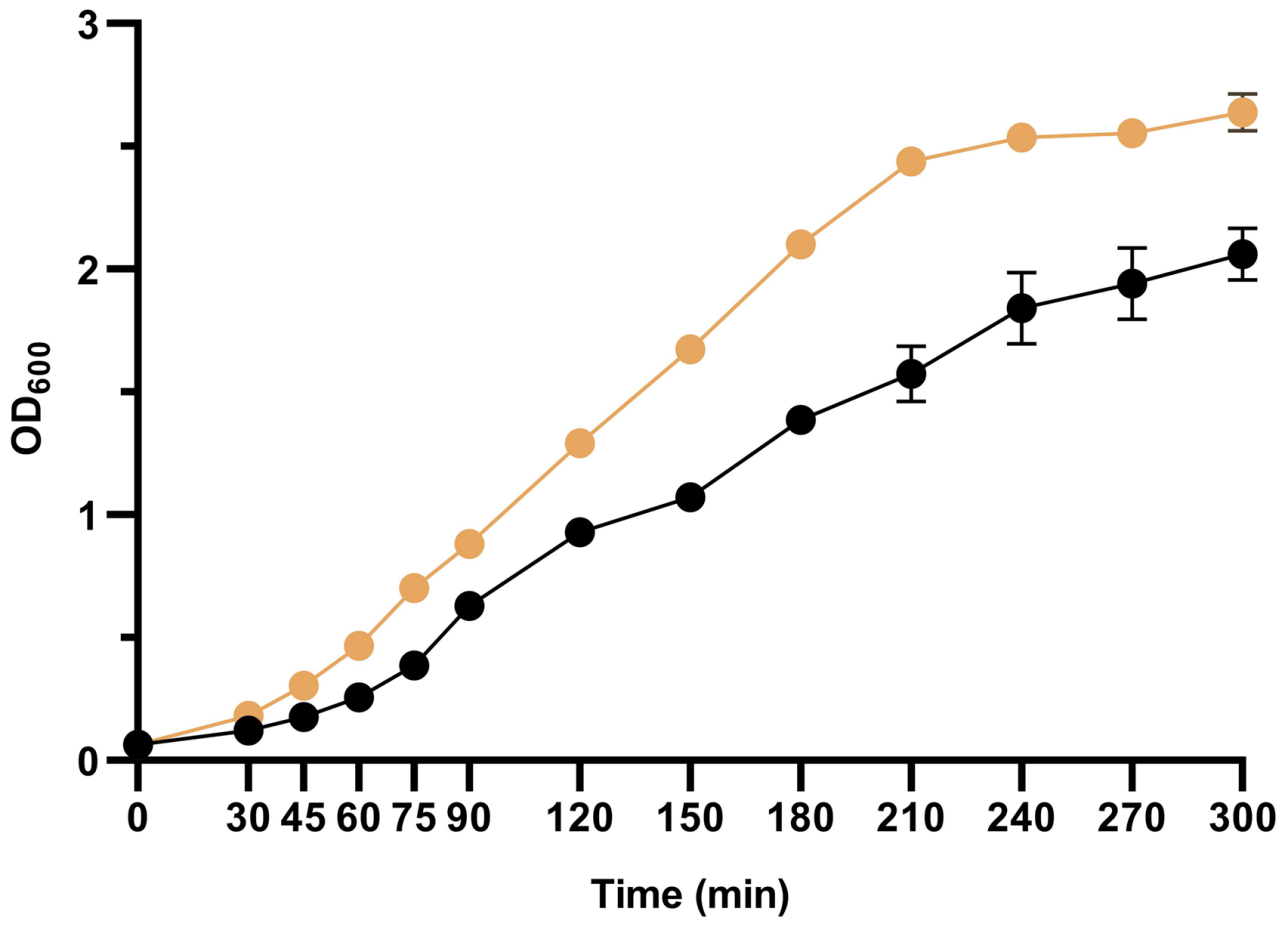
Growth of *E. coli* in liquid medium under standard gravity and simulated microgravity. An overnight *E. coli* culture grown under standard conditions (37 °C, 250 rpm) was diluted 1:20 and distributed into tubes containing 2 mL each. Cultures were incubated either in the standard incubator (37 °C, 250 rpm, black circles) or in the 3D-clinostat (37 °C, dual-axis rotation at 3:2.73 rpm, ochre circles). At the indicated times, two tubes per condition were sampled and OD₆₀₀ was measured. Points represent the mean of two replicates; error bars indicate standard deviation.

As a preliminary step to the evolution experiment with bacteriophage Qβ, we compared the growth of *E. coli* in liquid medium at 37 °C under two conditions: the routine laboratory protocol (250 rpm in the standard incubator; see Section 2.2) and in the 3D-clinostat (see Section 2.1). The results showed that *E. coli* grew faster in the clinostat than in the standard incubator (Figure 3). However, this difference cannot be attributed solely to the simulated microgravity, as other factors may also contribute. In particular, while tubes in the standard incubator remain in a fixed vertical position, the clinostat continuously alters their orientation, likely increasing aeration due to greater surface exposure. Since *E. coli* growth is sensitive to oxygen availability, this could affect not only bacterial replication but also their physiological state and susceptibility to phage infection.

To avoid this confounding factor, we chose to perform infections in semisolid agar in Petri dishes, where the surface exposed to air remains constant across conditions. In this setup, bacterial cells are largely immobilized, and the ability of the phage to diffuse through the agar becomes a key determinant of virus-host encounters, especially during the early stages of infection. While *E. coli* can exhibit motility in semisolid media through swimming or swarming, these behaviors are strongly dependent on agar concentration and cell density. Swimming is typically observed at agar concentrations ≤ 0.3% (Croze et al., 2011), whereas swarming requires a lag phase of 2–4 h, during which cells accumulate and remodel their physiology in response to surface contact and density cues (Partridge, 2022). In our experiments, the top agar concentration was 0.7%, and infections were incubated for only 2 h, a timeframe that precedes the onset of swarming and limits bacterial movement. Therefore, we consider phage diffusion to be a critical factor influencing infection dynamics under these conditions. This constraint on phage mobility may also influence the selective pressures acting during serial propagation, as discussed in later sections.

### 3.2 Reduced replication efficiency of Qβ under simulated microgravity

**Figure 4:**
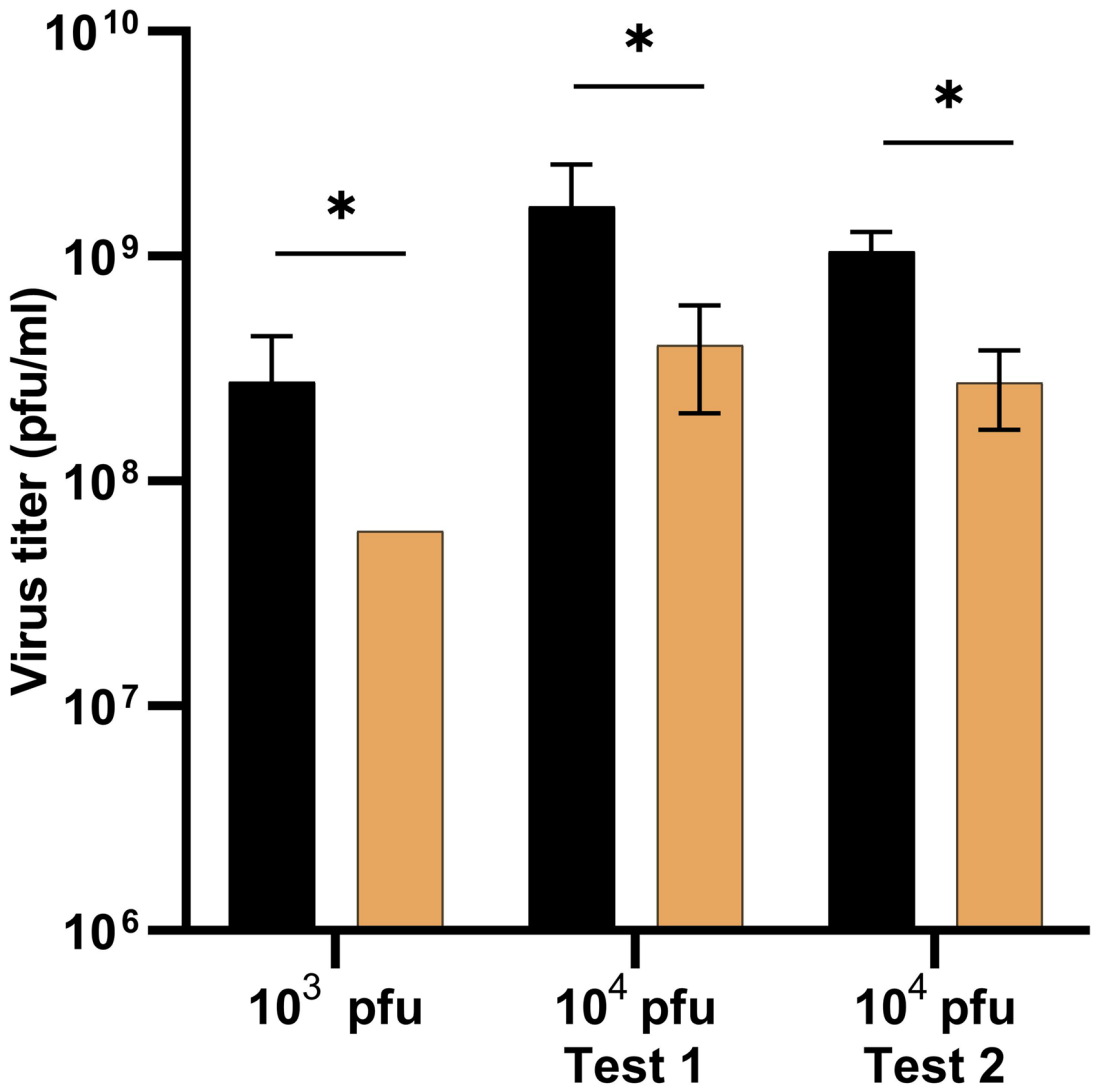
Virus titers obtained in Qβ infections carried out in semisolid agar either in simulated microgravity (3D-clinostat) or in normal gravity (standard incubator). The results of three different assays are represented, one in which infections were carried out with 10^3^ pfu and two others with 10^4^ pfu. Bacteria used for infections had been previously grown in the standard incubator until reaching an OD_600_ ≅ 0.8. Each infection assay was carried out in triplicate both in the standard incubator (black bars) and in the 3D-clinostat (ochre bars). Each bar represents the mean of the three values obtained for each condition. Standard deviations are also depicted. Asterisks indicate that the differences between the two conditions tested in each experiment are statistically significant (*p* < 0.05; Mann–Whitney test).

To assess the impact of simulated microgravity on phage replication in semisolid agar, we performed infections with the virus Qβ_Anc_ using two different viral loads: 10^3^ and 10^4^ pfu. The initial experiment with 10^3^ pfu served as a pilot to evaluate whether simulated microgravity affected viral yield. Upon observing a significant reduction in titers (Figure 4), we conducted two additional experiments with 10^4^ pfu to test the reproducibility of the effect and to determine whether the inhibitory impact of simulated microgravity was dependent on the initial viral load. The results (Figure 4) confirmed that replication efficiency was significantly reduced under simulated microgravity conditions (*p* < 0.05; Mann–Whitney test) and that the effect was consistent across different viral inputs.

### 3.3 Qβ evolution under simulated microgravity conditions

The results shown in Figure 4 indicate that simulated microgravity reduces the yield obtained in Qβ infections. To investigate whether the phage could develop adaptive responses to this selective pressure, we designed an evolution experiment in which Qβ was propagated in semisolid medium for 10 serial transfers (Figure 2). At each transfer, infections were performed either in normal gravity (standard incubator; S) or in simulated microgravity (3D-clinostat; C). Bacteria used for infections had been previously grown in liquid medium either in the standard incubator or in the 3D-clinostat until reaching an OD_600_ ≅ 0.8. This design allowed us to test four evolutionary scenarios, combining the conditions under which bacteria were grown and infections were carried out: SS (bacteria grown and infected in the standard incubator), SC (bacteria grown in the standard incubator and infected in the clinostat), CS (bacteria grown in the clinostat and infected in the standard incubator), and CC (bacteria grown and infected in the clinostat). It is worth noting that this experimental design permits only the evolution of phages, as fresh bacteria are introduced in each transfer.

**Figure 5:**
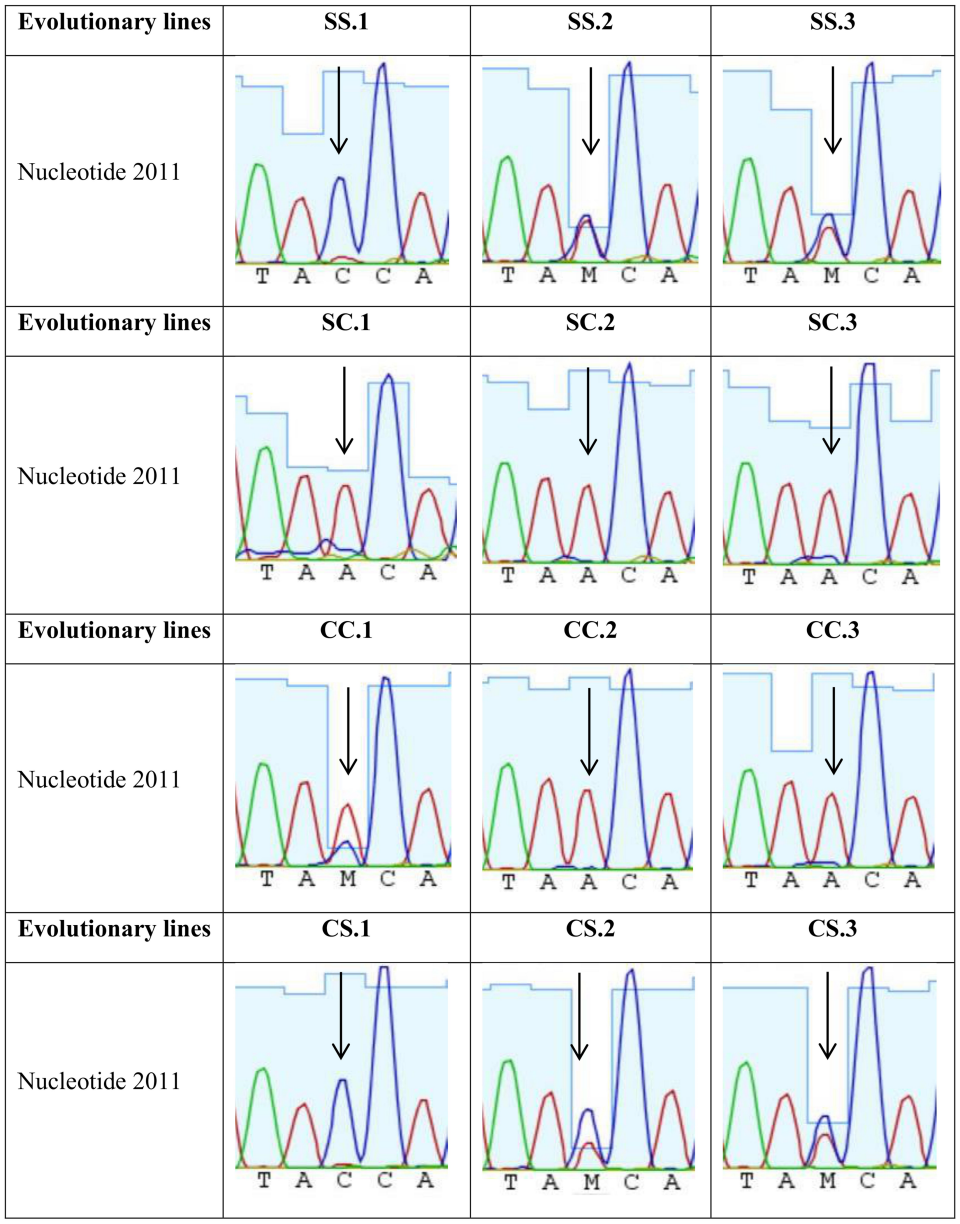
Chromatograms corresponding to the nucleotide position 2011 of the Qβ genome in the evolutionary lines tested. The evolutionary lines correspond to those described in the legend of Figure 2 and in Section 2.4 of Materials and methods. The arrow points to the 2011 position in the Qβ genome, showing the nucleotide (or mixture of nucleotides) present in each line.

Determination of the consensus sequences of the viral populations at transfer 10 revealed a single mutation, C2011A (resulting in the amino acid substitution T222N in the A1 protein), in some of the evolved lines (Figure 5). The mutation was fixed in all six lines where infections were performed in the 3D-clinostat (lines SC and CC), regardless of the previous bacteria growth condition. In contrast, the mutation was not fixed in any of the lines evolved in the standard incubator (lines SS and CS). It was undetectable in two of them and present as a polymorphism, reaching at most 50%, in the remaining four.

Mutation C2011A had previously been identified in our laboratory as beneficial for replication in well-mixed liquid cultures with low bacterial density (Laguna-Castro and Lázaro, 2022). In the current experiment, the concentration of bacteria in the semisolid agar was always around 2 × 10^7^ colony forming units per ml (cfu/mL), a condition favorable for the selection of the mutation. However, its fixation in all the lines evolved in the clinostat, whereas it was absent or remained as a polymorphism in the lines evolved in the standard incubator suggests that its selective value is greater under simulated microgravity.

**Figure 6:**
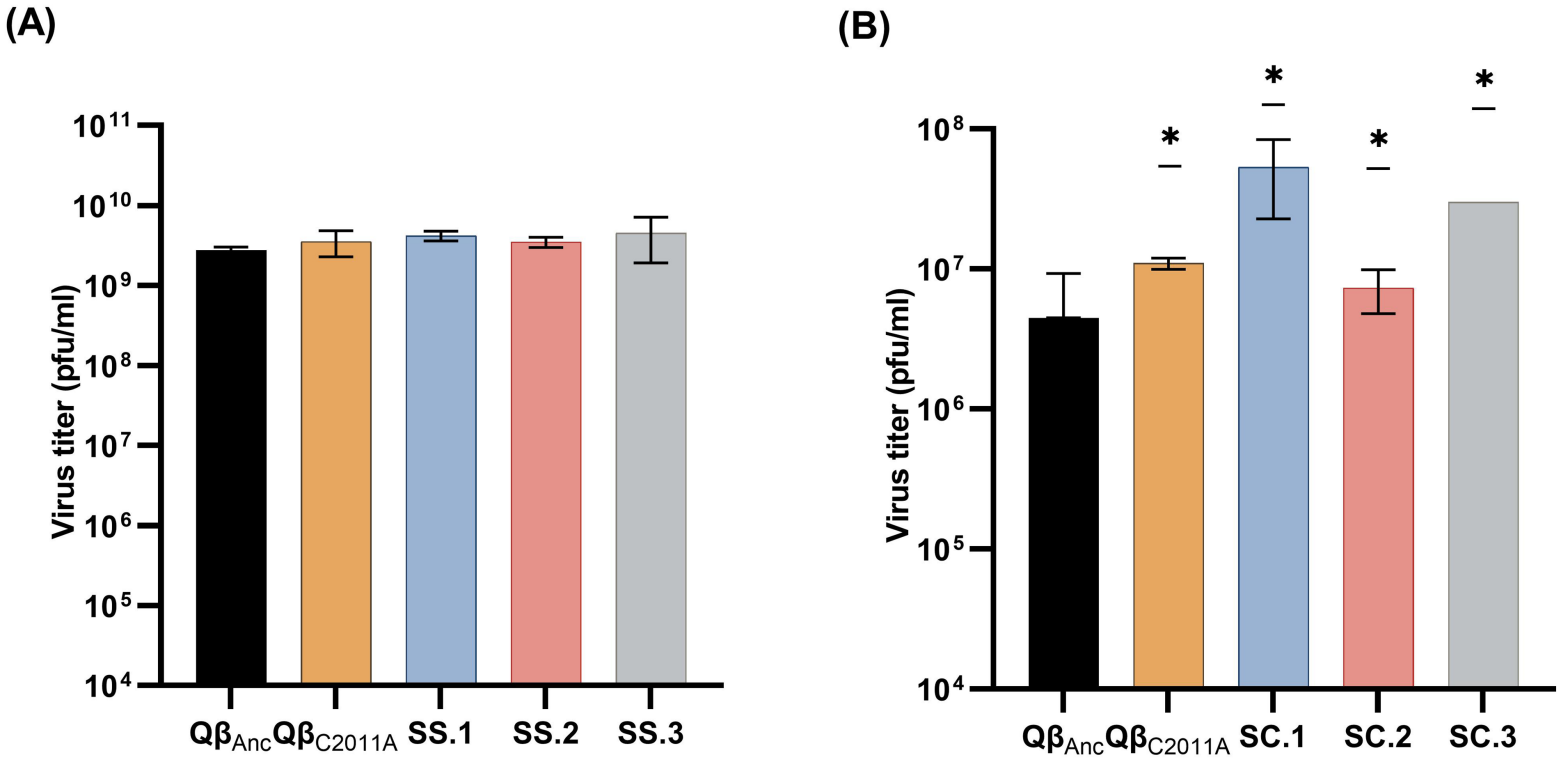
Virus titers obtained in semisolid agar when infecting with lines SS and SC and the viruses Qβ_Anc_ and Qβ_C2011A_. **(A)** Assay carried out in the standard incubator with lines SS. **(B)** Assay carried out in the 3D-clinostat with lines SC. Bacteria used for infections had been previously grown in the standard incubator until reaching an OD_600_ ≅ 0.8. Each bar represents the mean of three values obtained for each virus or evolutionary lines assayed. Standard deviations among replicas are also depicted. Asterisks indicate that the difference with the ancestor is statistically significant (*p* < 0.05; Mann–Whitney test). See Section 2.5 for further details.

To assess whether the evolved lines were optimized with respect to the ancestor, we performed replication assays using lines SC and SS. These were chosen because the bacterial growth condition prior to infection did not appear to influence the selection of mutation C2011A (Figure 5). The results showed that SS lines did not differ significantly from the ancestor in infections carried out in the standard incubator (*p* > 0.05; Mann–Whitney test) (Figure 6A), while SC lines exhibited significantly higher viral titers in infections carried out in the 3D-clinostat (*p* < 0.05; Mann–Whitney test) (Figure 6B).

Although the only mutation we detected in the consensus sequences of the evolved populations was C2011A, this does not exclude the presence of additional low-frequency mutations, which altogether could contribute to the increase in the replicative fitness of the lines evolved in the 3D-clinostat. To study the effect of mutation C2011A when present in a simpler mutant spectrum, we used the single mutant Qβ_C2011A_. It was observed that the viral titers of the mutant did not differ significantly from those of the ancestor when replication was performed in the standard incubator (*p* > 0.05; Mann–Whitney test) (Figure 6A), whereas they were significantly higher when replication took place in the 3D-clinostat (*p* < 0.05; Mann–Whitney test) (Figure 6B).

### 3.4 C2011A mutation favors an earlier start of infections in simulated microgravity conditions

The fact that mutation C2011A enhances viral entry into *E. coli* cells (Laguna-Castro and Lázaro, 2022) explains its selective advantage in infections with low bacterial density, such as those performed during the evolution experiment shown in Figure 2. In addition, in semisolid agar infections, where bacterial cells are largely immobilized (see Section 3.1), phage diffusion becomes another limiting factor for initiation of infections. This constraint likely increases the selective pressure for mutations that improve entry efficiency once a virus-bacterium encounter occurs. In the present study, mutation C2011A was detectable in 10 out of the 12 evolved lines, all propagated in semisolid medium. However, it was only fixed in the lines evolved under simulated microgravity, suggesting that this condition imposes additional barriers to the infection, thereby amplifying the selective advantage of the mutation.

**Figure 7:**
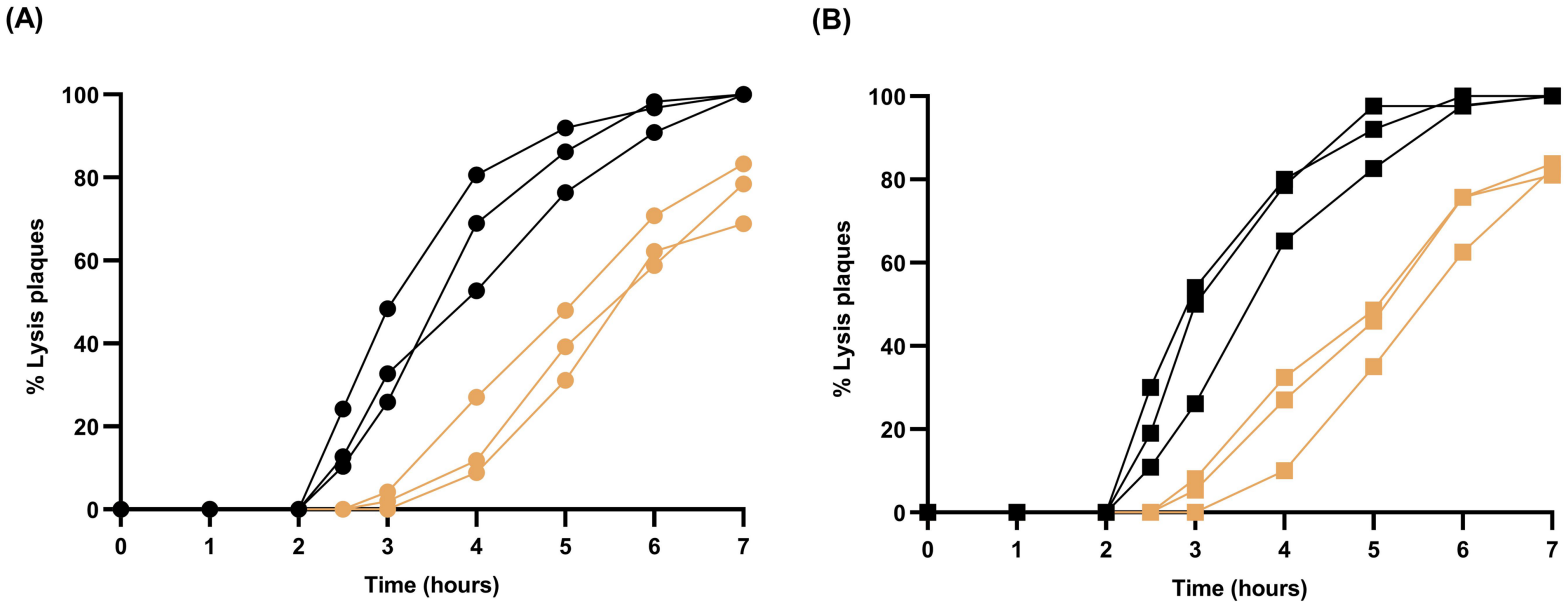
Kinetics of lysis plaque formation. **(A)** Virus Qβ_Anc_. **(B)** Virus Qβ_C2011A._ Each curve represents the results obtained in a Petri dish. In all cases, the percentage of plaques at each time was calculated with respect to the total obtained in the same Petri dish at 24 h. In both panels, black symbols refer to infections carried out in the standard incubator and ochre symbols to infections in the 3D-clinostat. See Section 2.6 for additional details.

If it is indeed more difficult for the phage to initiate infections under simulated microgravity conditions, lysis plaques would be expected to appear later or even occur in smaller numbers in the 3D-clinostat than in the standard incubator. To test this possibility, we performed an experiment in which bacteria were infected using low viral loads of either the ancestral virus (≅ 60 pfu) or the mutant Qβ_C2011A_ (≅ 50 pfu). Petri dishes were incubated in the standard incubator or in the 3D-clinostat, and lysis plaques were counted at various time points (Figure 7). It is noticeable that, for the same virus, the curves obtained when infections were carried out in the standard incubator were always above those obtained for infections performed in the 3D-clinostat. In addition, although the difference was not statistically significant (*p* > 0.05; Mann–Whitney test), the total number of plaques observed at 24 h was higher in the standard incubator (58.3 ± 3.5 for Qβ_Anc_ and 46.0 ± 4.0 for Qβ_C2011A_) than in the 3D-clinostat (48.0 ± 3.0 for Qβ_Anc_ and 38.0 ± 1.7 for Qβ_C2011A_). Although the mutant appeared to initiate infections slightly earlier than the ancestral virus (compare Figures 7A,B), the effect was quite subtle and difficult to interpret given variability among replicates. Because data were normalized to the total plaques observed at 24 h, relative dynamics can be compared, but conclusions remain limited by the modest magnitude of the differences.

**Figure 8:**
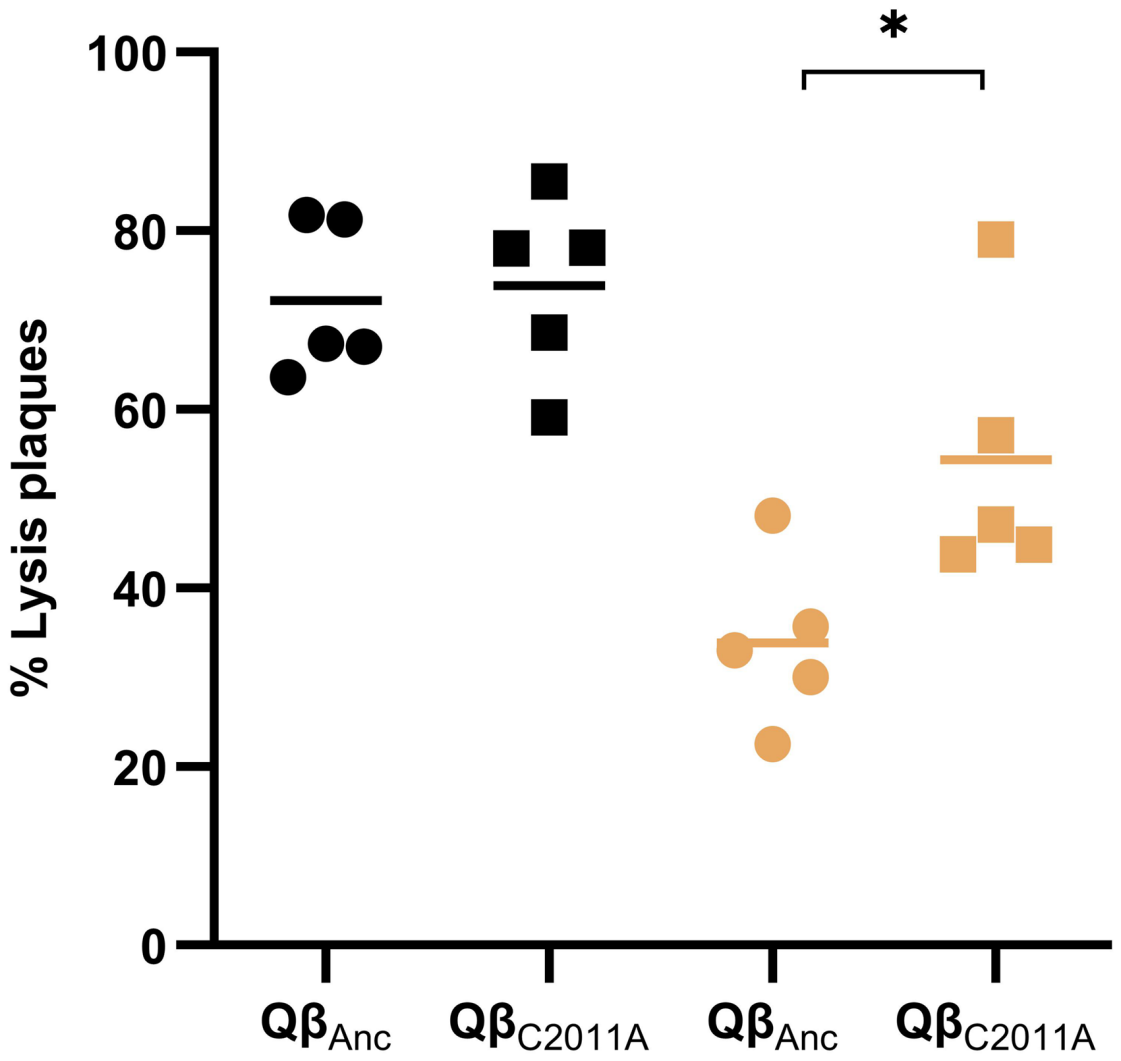
Percentage of lysis plaques observed after 4 h of incubation. Values are expressed relative to the total number of lysis plaques observed at 24 h, in infections performed with either Qβ_Anc_ or Qβ_C2011A_, under standard gravity (black symbols) or simulated microgravity (3D-clinostat; ochre symbols). Each data point represents the result from an individual Petri dish. Horizontal lines indicate the mean values obtained from five replicates per condition. The asterisk denotes statistically significant differences (*p* < 0.05; Mann–Whitney test). See Section 2.6 for additional details.

To address the limitations of the previous assay and assess whether mutation C2011A affects infection onset, we performed a complementary experiment (Figure 8). Both the ancestral virus and the mutant Qβ_C2011A_ were tested under standard gravity and simulated microgravity conditions. As in Figure 7, the number of plaques observed after 4 h was normalized to the total plaques at 24 h, enabling direct comparison of early infection dynamics. No significant differences were detected between the two viruses in the standard incubator (p > 0.05; Mann–Whitney test). In contrast, under simulated microgravity, the mutant produced significantly more plaques than the ancestor (*p* < 0.05; Mann–Whitney test), indicating that mutation C2011A facilitates earlier infection initiation in this environment.

Although the viral load used for Qβ_C2011A_ (≅ 120 pfu) was slightly higher than that used for the ancestral virus (≅ 50 pfu) in this experiment, this difference is minor in the context of plaque assays, where viral suspensions are typically diluted from stocks containing up to 10^10^ pfu/ml. Moreover, because the data were normalized to the total number of plaques observed at 24 h, the comparison of infection onset dynamics remains valid. The earlier appearance of plaques in the mutant virus under simulated microgravity is consistent with previous findings (Laguna-Castro and Lázaro, 2022), where mutation C2011A was shown to enhance viral entry under conditions of low bacterial density. This advantage is expected to be particularly relevant in semisolid agar, where phage diffusion is limited and initial virus-host encounters are less frequent.

The earlier onset of infections in the standard incubator than in the clinostat suggests that the differences in viral titers could be greater if the incubation time of the bacteria with the virus was shorter. To test this idea, we compared the virus titers obtained after 1 and 2 h in triplicate infections performed in semisolid agar with the virus Qβ_Anc_ and the mutant Qβ_C2011A_. While titers at 2 h were 7.5 times higher in the standard incubator than in the clinostat, this difference increased to 25-fold at 1 h (data not shown), reinforcing the idea that delayed infection initiation is a key factor limiting viral replication under simulated microgravity.

## 4 Discussion

This study explores the effects of simulated microgravity on the short-term evolution of the RNA bacteriophage Qβ, which is probably a normal component of the human gut microbiome where it infects the bacteria *E. coli* (Arisaka, 2024). The good previous knowledge that exists about this phage and its evolution in different conditions (Bradwell et al., 2013; Lázaro et al., 2018; Somovilla et al., 2019; Laguna-Castro et al., 2023, 2024) makes it an excellent model to investigate how bacteria-phage interactions could be affected in space conditions.

Our results show a significantly lower yield of Qβ infections carried out in simulated microgravity conditions compared to those performed in normal gravity, regardless of the initial viral load used for infection (Figure 4). This observation aligns with previous studies indicating that microgravity (real or simulated) can disrupt biological processes such as molecular transport and macromolecular assembly (Chang et al., 1993; McPherson and DeLucas, 2015; Koaykul et al., 2019; Zhou et al., 2020; Martirosyan et al., 2022; Lehr et al., 2024), which are critical for efficient viral replication. In addition, in semisolid agar infections, such as those carried out in this work, bacteria and phages encounters are mainly determined by the diffusion capacity of the phage, which can also be affected by gravity conditions. The lower the ability of the phage to spread, the less likely it is to find a susceptible bacterium, which probably translates into a delay in the average time needed to initiate an infection, giving rise to lower viral titers. The results showing a later onset of infections under simulated microgravity in the 3D-clinostat (Figures 7, 8) could be well explained on the basis of a decreased phage diffusion rate under this condition. This finding underlines the importance of diffusion dynamics in the success of phage infections in structured mediums, such as biofilms formed by many bacteria, including those present in the human gut, and suggests that microgravity might enhance the selective pressure to optimize the process.

The evolution experiment we have carried out revealed that Qβ was capable of improving its replication in the 3D-clinostat within a relatively short timeframe. Mutation C2011A, which introduces an amino acid substitution (T222N) in the A1 protein, was fixed in all evolutionary lines propagated under simulated microgravity, whereas it remained polymorphic or absent in the lines evolved under normal gravity (Figure 5). Previous work in our laboratory identified this mutation as advantageous for virus replication in low-density bacterial cultures carried out in liquid medium with good shaking (Laguna-Castro and Lázaro, 2022). We also demonstrated that the mutation increases virus entry into bacteria, which may also make it of selective value when replication takes place on semisolid agar, a medium in which bacteria-phage encounters are hindered by the limited mobility of bacteria and the constrained diffusion of phages.

Although the mutation was not exclusive to the clinostat-evolved lines, its consistent fixation under simulated microgravity suggests that the selective pressure in that environment may have been stronger. We acknowledge that the result would have been more compelling had the mutation been entirely absent from the standard incubator lines. However, it is important to note that adaptive mutations do not necessarily need to be specific to a single environment. A mutation may confer a general advantage under multiple conditions, with its fixation rate varying depending on the intensity of the selective pressure. In our case, mutation C2011A enhances viral entry under low bacterial density (Laguna-Castro and Lázaro, 2022), a condition that inherently reduces the likelihood of successful infection. This advantage is likely relevant in semisolid agar, where initial virus-host encounters are limited, and even more so under simulated microgravity, where diffusion constraints may be exacerbated due to the constantly changing gravity directionality. This layered selective context likely explains the accelerated fixation of C2011A in the clinostat lines. Future deep sequencing of the evolved populations could help identify additional low-frequency mutations contributing to adaptation and clarify the specific selective pressures acting in each environment.

This reasoning is supported by the observation that the mutant virus Qβ_C2011A_ exhibited significantly higher replication rates than the ancestral virus in the clinostat but not in the standard incubator (Figure 6). The findings are consistent with previous results obtained in the T7 bacteriophage and *E. coli* system, where microgravity conditions were shown to drive the emergence of mutations in both the phage and the host, enhancing their capacity for interaction (Huss et al., 2023). Such parallels strengthen the notion that microgravity exerts distinctive selective pressures on virus–host systems, where even broadly adaptive mutations may become preferentially fixed due to the cumulative constraints of the environment.

The earlier onset of lysis plaques observed in standard gravity conditions compared to simulated microgravity (Figure 7) further underscores the impact of gravity on the kinetics of phage infections. The delay in plaque formation in the clinostat indicates that initiation of infections takes longer in this condition than in the standard incubator. Mutation C2011A appears to mitigate this effect, as evidenced by the higher number of lysis plaques formed at early times by the virus Qβ_C2011A_ compared to the ancestor when infections are carried out in the 3D-clinostat (Figure 8). The fact that viral titers experienced a sharper decline in the clinostat with respect to the standard incubator when the incubation time with bacteria was shorter (1 h versus 2 h) also points to the later onset of infections as the main factor affecting the reduction of viral titers under simulated microgravity conditions.

Another factor that could influence bacteriophage Qβ replication under the conditions tested is the physiological state and growth capacity of *E. coli* (Zea et al., 2017). It is described that many bacteria undergo alterations when grown under microgravity conditions, both in ground-based simulations and spaceflight experiments (Nickerson et al., 2004; Rosenzweig et al., 2010; Rea et al., 2016; Huang et al., 2018; Santomartino et al., 2020; Acres et al., 2021; Cortesão et al., 2022). In our preliminary tests, *E. coli* grew faster in the 3D-clinostat than in the standard incubator (Figure 3), consistent with previous reports showing that simulated microgravity can accelerate growth and modulate gene expression (Arunasri et al., 2013; Topolski et al., 2022). However, this difference may also reflect increased aeration in the clinostat, where tube orientation changes continuously, exposing a larger surface to air. Because oxygen availability strongly affects *E. coli* growth, subsequent phage infections were performed on semisolid agar to maintain a constant exposed surface.

Until now, we have stated that mutation C2011A favors phage replication in simulated microgravity because, under this condition, phage diffusion is slower, meaning that a mutation that favors virus entry once the encounter with the bacteria has occurred is advantageous. An alternative possibility is that bacteria could express fewer receptors for the phage, F pili in this case, when grown under simulated microgravity conditions (Piscon et al., 2023). In such a scenario, the selective value of mutation C2011A would also be enhanced. Some preliminary experiments we have conducted do not show appreciable differences in virus entry in bacteria grown in liquid medium in the clinostat or the standard incubator. However, it would be necessary to carry out more precise studies to determine if the expression of the Qβ receptor in *E. coli* is altered in simulated microgravity.

The ability of Qβ to adapt to simulated microgravity has broader implications for understanding phage-bacteria interactions in extraterrestrial environments. Given the essential role of bacteriophages in regulating microbial ecosystems, changes in phage dynamics could influence the composition and stability of microbial communities in space habitats. This is particularly relevant for human health during long-term space missions, as disruptions in the gut microbiome - partly regulated by phages - could have significant physiological consequences. Furthermore, the observation that simulated microgravity can alter the selective pressures experienced by viruses emphasizes the need for more comprehensive studies on microbial evolution in space environments.

While we acknowledge that simulated microgravity using clinostats does not perfectly replicate true microgravity, we believe our findings are quite valuable for future research that helps elucidate how variations in gravitational conditions impact phage-bacteria interactions in space and extraterrestrial environments. The findings shown contribute to our understanding of life adaptation and underscore the importance of considering microgravity as a key factor in the design of space missions and planetary protection protocols. Future experiments conducted in true microgravity environments, such as aboard the ISS, could validate and expand the conclusions drawn here, providing deeper insights into the complex interplay between gravitational forces and biological systems.

## Data Availability

The original contributions presented in the study are included in the article/Supplementary material, further inquiries can be directed to the corresponding author.

## Footnotes

1
https://www.geneious.com
