# Supplementary material for: Impact of simulated microgravity in short-term evolution of an RNA bacteriophage
Source: Front Microbiol. 2025 Dec 4;16:1680651. doi: 10.3389/fmicb.2025.1680651 (PMC12712649; doi:10.3389/fmicb.2025.1680651)
Supplement: Supplementary file 1 [file Data_Sheet_1.docx]

Supplementary Material

# Supplementary Figures


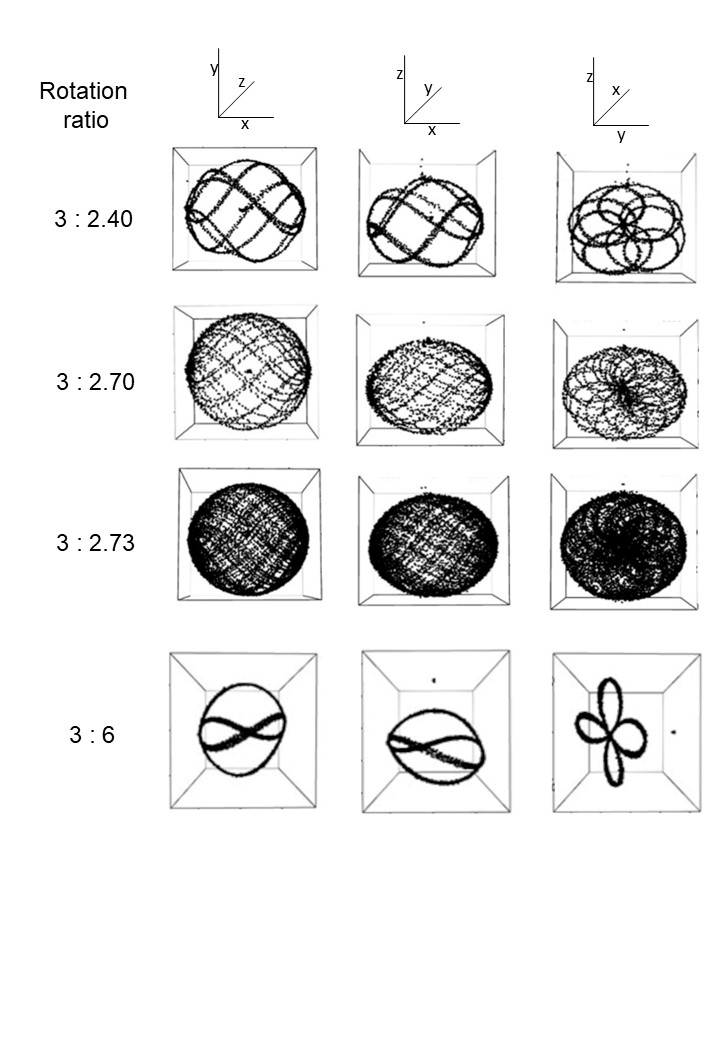
**Supplementary Figure 1**. 3D-Clinostat trajectories under different rotation ratios (first axis : second axis; rpm values). A final ratio of 3 : 2.73 was selected for microgravity simulation experiments.
